# Functional validation of the novel KIF5A p.R17Q VUS reveals defective axonal transport in iPSC-motoneurons from a SPG10 patient

**DOI:** 10.3389/fgene.2026.1774170

**Published:** 2026-02-27

**Authors:** Serena Santangelo, Valeria Casiraghi, Claudia Fallini, Sabrina Invernizzi, Silvia Peverelli, Martina Bertocchi, Monica Feole, Marta Cozzi, Stefania Magri, Angelo Poletti, Patrizia Bossolasco, Franco Taroni, Vincenzo Silani, Antonia Ratti

**Affiliations:** 1 Department of Medical Biotechnology and Translational Medicine, Università degli Studi di Milano, Milan, Italy; 2 Department of Cell and Molecular Biology, Ryan Institute for Neuroscience, University of Rhode Island, Kingston, RI, United States; 3 Department of Neuroscience - Laboratory of Neuroscience, Milan, Italy; 4 The Board of Governors Regenerative Medicine Institute, Cedars-Sinai Medical Center, Los Angeles, CA, United States; 5 Dipartimento di Scienze Farmacologiche e Biomolecolari “Rodolfo Paoletti”, Dipartimento di Eccellenza 2018-2027, Università degli Studi di Milano, Milan, Italy; 6 Unit of Medical Genetics and Neurogenetics, Fondazione IRCCS Istituto Neurologico Carlo Besta, Milan, Italy; 7 “Dino Ferrari” Center, Department of Pathophysiology and Transplantation, Università degli Studi di Milano, Milan, Italy

**Keywords:** iPSC, KIF5A, motor neurons, SPG10, VUS, axonal transport

## Abstract

Cytoskeletal alterations and axonal transport deficits are key factors in many neurodegenerative disorders. The neuronal kinesin family member 5A (KIF5A) is a microtubule-based motor protein critical for anterograde transport of RNA granules, organelles, and neurofilaments along axons and dendrites. Heterozygous missense and nonsense mutations in the N-terminal motor and stalk domains are associated with hereditary spastic paraplegia 10 (SPG10) and Charcot-Marie-Tooth disease type 2 (CMT2), while frameshift mutations in *KIF5A* C-terminal cargo-binding domain are linked to amyotrophic lateral sclerosis (ALS). We recently reprogrammed an iPSC line from a SPG10 patient carrying the novel missense variant c.50G>A (p.R17Q) in the *KIF5A* motor domain, classified as variant of unknown significance (VUS) and predicted to affect ATP binding. Here we gene-edited this mutant iPSC line by CRISPR-Cas9 to obtain an isogenic wild-type (WT) *KIF5A* cell line. We next examined functionally the impact of the p.R17Q VUS on KIF5A protein sub-cellular distribution and on axonal transport of mitochondria and lysosomes in differentiated iPSC-motoneurons (MNs). The presence of neurofilament-positive axonal swellings and an increased distribution of KIF5A protein in distal neurites was observed in the mutant p.R17Q compared to the WT *KIF5A* iPSC-MNs, indicating a likely defective axonal transport. The anterograde velocity and distance travelled by mitochondria and lysosomes along neurites was indeed significantly reduced in the mutant *KIF5A* iPSC-MNs compared to the WT ones. These findings demonstrate that the p.R17Q VUS is pathogenic, thereby extending the spectrum of *KIF5A* mutations causing SPG10 and support the use of patient-derived iPSC-MNs to functionally validate *KIF5A*-associated VUS.

## Introduction

Axonal transport defects are a well-known hallmark of neurodegenerative diseases (Chevalier-Larsen and Holzbaur, 2006; Guo et al., 2020). Kinesins, a large family of over 40 ATP-dependent motor proteins, play key roles in processes like intracellular transport and cell division (Hirokawa and Tanaka, 2015). In particular, Kinesin-1 (also known as KIF5) subfamily comprises three members (KIF5A, KIF5B, and KIF5C), where KIF5A and KIF5C are neuron-specific and involved in the anterograde axonal transport of cargoes including proteins (i.g. neurofilaments), RNA granules, and organelles (i.e. mitochondria and lysosomes) (Ali and Yang, 2020).

KIF5 members work as dimers with an N-terminal motor domain that binds microtubules and drives ATP hydrolysis, a central stalk region that mediates dimerization and interactions, and a C-terminal tail that regulates cargo binding, microtubule sliding and autoinhibition through an isoleucine-alanine-lysine (IAK) motif to prevent movement without cargoes (Miki et al., 2005; Baron et al., 2022).

Heterozygous gene variants targeting the three KIF5A domains are linked to distinct neurodegenerative or neurodevelopmental disorders (Cozzi et al., 2025). Missense and nonsense mutations in KIF5A motor and stalk domains are associated with a form of hereditary spastic paraplegia (SPG10, Online Mendelian Inheritance in Man (OMIM) #604187) (Reid et al., 2002; Jennings et al., 2017) and axonal Charcot-Marie-Tooth Type 2 disease (CMT2) (Crimella et al., 2012), while frameshift mutations in its C-terminal tail are linked to amyotrophic lateral sclerosis (ALS) (OMIM #617921) (Brenner et al., 2018; Nicolas et al., 2018) and neonatal intractable myoclonus (NEIMY, OMIM #617235) (Duis et al., 2016).

However, the underlying biological mechanisms and functional alterations leading to this wide spectrum of *KIF5A*-related diseases still remain poorly understood. To address this issue, we recently reported the functional characterization of five selected rare variants associated with different *KIF5A*-linked phenotypes - SPG10 (p.R17Q, p.R280C), CMT2 (p.R864*), NEIMY (p.C975Vfs*73), and ALS (p.N999Vfs*40) - which we identified through *KIF5A* genetic testing in a large cohort of ∼2,150 Italian index cases (Cozzi et al., 2024). In particular, mutant KIF5A protein subcellular localization and effects on mitochondria distribution and proteasome system were analyzed in murine motoneuron-like NSC-34 cells and in human neuroblastoma cells over-expressing all these five variants, including the novel p.R17Q one (Cozzi et al., 2024).

In more recent years, the use of human induced pluripotent stem cells (iPSCs) has enabled the functional investigation, in a more physiological context, of *KIF5A* mutations in motoneuron-differentiated cells. While studies in iPSC-derived motoneurons (iPSC-MNs) carrying ALS-linked mutations have reported changes in KIF5A protein distribution, autoinhibition regulatory mechanism and axonal transport rates (Baron et al., 2022; Pant et al., 2022), no functional evidence of SPG10-linked *KIF5A* variants have been reported so far but in mouse and *Drosophila* animal models (Wang and Brown, 2010; Füger et al., 2012). We recently generated an iPSC line from a patient belonging to a SPG10 family in which the novel missense variant c.50G>A (p.R17Q) segregated with the disease in 3 affected members (Santangelo et al., 2023). The p.R17Q variant was classified as variant of unknown significance (VUS) and predicted to map in the ATP-binding pocket of the KIF5A motor domain (Cozzi et al., 2024).

We here generated the isogenic wild-type (WT) *KIF5A* iPSC line using CRISPR-Cas9-based gene-editing from the patient-derived mutant p.R17Q cell line to functionally evaluate its possible pathogeneicity in SPG10. We investigated the biological effects of this VUS by analyzing mutant KIF5A protein distribution and axonal transport of mitochondria and lysosomes in iPSC-MNs.

## Methods

### Gene-editing by CRISPR/CAS9

We gene-edited the iPSC line previously generated from the SPG10 patient carrying the novel *KIF5A* variant (c.50G>A, p.R17Q, NM_004984.4) (Santangelo et al., 2023).

The web resource https://www.idtdna.com/site/order/designtool/index/CRISPR_SEQUENCE was employed to design the single-guide RNA (sgRNA) sequenceTCAAGGTGCTCTGCCGATTCCGG, targeting the region in the first exon of *KIF5A* gene containing the c.50G>A single nucleotide variant. The same web resource by IDT (above) and an additional software by Wellcome Sanger Institute Genome Editing (https://wge.stemcell.sanger.ac.uk/find) were used to check and exclude high probability off-target sites in coding sequences in the first 10 hits (Supplementary Table S2).

The following Alt-R single-stranded oligodeoxynucleotide (ssODN) sequence was used to introduce the WT *KIF5A* sequence (c.50G): AGA​CCA​ACA​ACG​AAT​GTA​GCA​TCA​AGG​TGC​TCT​GTC​GAT​TCC​GGC​CCC​TGA​ACC​AGG​C TGA​GAT​TCT​GCG​GGG​AGA​CAA​GTT​C.

For the iPSC gene-editing, we adapted the protocol provided by IDT company. Briefly, 8 × 10^5^ iPSCs were transfected with a ribonucleoprotein (RNP) complex containing synthetic sgRNA, Alt-R HiFi Cas9 nuclease V3, Electroporation Enhancer and Alt-R ssODN (all from IDT) using the Amaxa nucleofector II system. Cells were then plated on Synthemax II-SC (Corning)-coated wells in StemFlex medium (Life Technologies) supplemented with the Homology Directed Repair (HDR) enhancer (IDT), incubated at 32 °C for 48 h and then at 37 °C. On day 11, the grown colonies were dissociated, clonally diluted, and transferred to multi-well plates for expansion and subsequent Sanger sequencing analysis.

### Sanger sequencing

Genomic DNA was extracted from iPSC pellets using Wizard® Genomic DNA Purification Kit (Promega). Amplicons encompassing the target *KIF5A* variant (c.50G>A) were obtained using specific primer pairs (Fwd: CAG​AGA​CTG​AGC​ACC​TGT​CCT​CC; Rev: GGG​GAA​GAG​GAT​GAA​GGA​TGA​GC) and sequenced with BigDye Terminator kit (Applied Biosystems) on an ABI 3500 Genetic Analyzer (Applied Biosystems).

### iPSC cultures and motoneurons differentiation

The two iPSC lines (WT and p.R17Q) were cultured in E8 medium (Thermo Fisher Scientific), splitted twice a week and cultured in 5% CO_2_, at 37 °C.

For motoneurons differentiation, iPSCs were grown in suspension for 21 days to generate embryoid bodies (EBs), that were then dissociated and cultured for additional 13 days on poly-D-lysine and laminin (Thermo Fisher Scientific) pre-coated multi-well plates to obtain iPSC-MNs, as previously described (Casiraghi et al., 2025). The efficiency of iPSC-MNs differentiation was assessed by quantitative PCR and immunofluorescence.

### Quantitative PCR (Q-PCR)

Total RNA was isolated using the TRIzol Reagent (Thermo Fisher Scientific) according to the manufacturer’s protocol, DNAse I-treated and then retro-transcribed using SuperScript II reverse transcriptase (Thermo Fisher Scientific) along with oligo-dT primers. Q-PCR was performed in technical duplicates for each sample using SYBR Green PCR Master Mix (Applied Biosystems) with 300 nM of specific primer pairs for 45 cycles on the QuantStudio 12K Flex system (Applied Biosystems). Specific primer pairs for iPSC stemness markers (*SOX2*, *OCT3/4*, and *NANOG*) and for iPSC-MNs characterization (*TUBB3*, *MAP2* and *ChAT*) were designed and used as previously detailed (Santangelo et al., 2023; 2024). Mean values of the threshold cycles (Ct) for each gene were normalized over mean Ct values of the housekeeping *RPL10A* gene (ΔCt). Each ΔCt was normalized over the ΔCt of the WT control sample (ΔΔCt) and gene expression values were expressed as fold change (2^−ΔΔCT^).

### Western blot (WB)

Cell pellets were resuspended in lysis buffer (20 mM Tris–HCl pH 7.5,150 mM NaCl, 1 mM EDTA, 1 mM EGTA, 1% Triton X-100, protease/phosphatase inhibitors cocktail) for 15 min at 4 °C, and protein content quantified by Bicinchoninic (BCA) method as previously described (Casiraghi et al., 2025). Protein lysates (30 µg) were resolved on precast 10% NuPAGE Bis-Tris polyacrilamide gels in 3-N-morpholino propanesulfonic acid (MOPS) buffer and then transferred onto nitrocellulose membranes (all from Thermo Fisher Scientific). Membranes were blocked with 5% Bovine Serum Albumin (BSA) in Tris-buffered saline (Santa Cruz Biotechnology) with Tween-20 (Sigma-Aldrich) (TBST) and incubated with the primary antibodies for KIF5A and α-Tubulin (Supplementary Table S1) in blocking solution at 4 °C overnight. The membrane was then washed 4 times with TBST for 5 min and incubated 1 h at room temperature (RT) with the appropriate HRP-conjugated secondary antibody (Supplementary Table S1). The Clarity™ Western ECL Substrate (Biorad) was used for signal detection and densitometric analyses were performed using the Quantity One software (Biorad).

### Immunofluorescence (IF)

IF was performed as previously described (Santangelo et al., 2024). Cells on coverslips were fixed using 4% paraformaldehyde (Santa Cruz Biotechnology) for 20 min at RT and permeabilized with ice-cold methanol and 0.3% Triton X-100 (Sigma-Aldrich) for 5 min each. Fixed cells were then incubated with 10% Normal Goat Serum (Gibco) in phosphate buffered saline (PBS) at RT for 20 min, followed by incubation with specific primary antibodies (Supplementary Table S1) at 37 °C for 1.5 h. Coverslips were then incubated with fluorescent labelled secondary antibodies (Supplementary Table S1) for 45 min at RT. Nuclei were counterstained with 4′,6-diamidino-2-phenylindole (DAPI) (Sigma-Aldrich).

To visualize mitochondria, live cells were incubated with 10 nM Mitotracker Red CMXRos (Thermo Fisher Scientific) for 15 min in Neurobasal medium (Thermo Fisher Scientific). Cells were washed twice with PBS and then fixed for IF.

### Image acquisition and analysis

Images were acquired on a Nikon Eclipse Ti confocal microscope at 40x or 60x as Z-stacks (0.5 µm step size) at a resolution of 1,024 × 1,024 pixels.

The ImageJ software (https://imagej.net/software/fiji/) was used for different image analyses:

- Mean fluorescence intensity (MFI): the multiple stacks of each channel were combined using the sum slices criterion. The “Rectangle” or “Free Hand” functions were used to select the regions of interest (ROI), whose fluorescence signal intensity was measured with the “Measure” function. KIF5A protein MFI was measured in the soma cytoplasm (2.5 × 2.5 μm), in neurite segments (10–20 μm long) proximal to the soma and in distal neurite segments (50–60 μm long) ∼50 μm far from the soma.
- Axonal swellings: the multiple stacks of each channel were combined using the maximum intensity criterion. The SMI-312 marker was used to both visualize and count the number of axonal swellings. We then measured the ratio between the number of axonal swellings and the number of DAPI-stained nuclei of iPSC-MNs positive for the SMI-312 marker in each field.
- Mitochondrial Aspect Ratio (AR): the multiple stacks of each channel were combined using the maximum intensity criterion. The “Segment line” function, with a width of 15 pixels, was used to select the segments of interest. The acquired images were binarized after setting the appropriate threshold, and mitochondria were analyzed using the “Shape Descriptors” plugin. The mitochondrial AR was determined as width to height ratio.

### Time-lapse imaging of mitochondria and lysosomes

The confocal microscope Nikon Eclipse Ti with a 20x and a ×40 objective was used for live imaging of mitochondria and lysosomes, respectively. The cell chamber conditions were set at 5% CO_2_, 37 °C, 95% Relative Humidity (RH). At day 21 of differentiation, EB-dissociated cells were plated on 35 mm glass-bottom dishes and cultured for 13 days to obtain iPSC-MNs as described above.

To image mitochondrial transport along neurites, iPSC-MNs were incubated with 10 nM Mitotracker Red CMXRos (Invitrogen) diluted in Neurobasal medium for 15 min at 37 °C. After replacement of the medium, cells were washed twice with PBS and then iPSC-MNs culture medium was added to image the mitochondria transport with 1 frame every 2 s for 3 min with a ×20 objective.

To image lysosomal axonal transport, iPSC-MNs were labelled with 50 nM LysoTracker Red DND-99 (Invitrogen) for 15 min at 37 °C. Cells were washed with PBS and incubated with artificial cerebrospinal fluid (aCSF) pH 7.4 solution (NaCl 121 mM, KCl 2.5 mM, CaCl2 2.2 mM, MgSO4 1 mM, NaHCO3 29 mM, NaH2PO4 0.45 mM, Na2HPO4 0.5 mM, Glucose 20 mM) for 15 min at 37 °C (Feole et al., 2024). Images were recorded every 2 s for 1 min with a ×40 objective.

Given the difficulty in discriminating between axons and dendrites in iPSC-MNs, we performed MN differentiation at low density culture condition (5,000 EB-dissociated cells) and we considered the longest neurite extending from the soma as the main axon in our transport analyses. Mitochondrial and lysosomal velocities were tracked along segments approximately 50 μm in length and located within 100 μm distance from the nucleus.

Representative kymographs were generated from the time-lapse images using ImageJ software to illustrate the directional movement. The ImageJ “MTrackJ” plugin was used to analyze the mean velocity (μm/second) and the distance (μm) travelled by mitochondria and lysosomes along the selected segments (Supplementary Figure S3).

### Statistical analysis

Statistical analysis was conducted using Graphpad Prism 9 software, using non-parametric Mann–Whitney U test. Results were considered statistically significant when p-value was <0.05.

## Results

### Generation and characterization of the isogenic WT line from the mutant SPG10-*KIF5A* iPSC line

**FIGURE 1:**
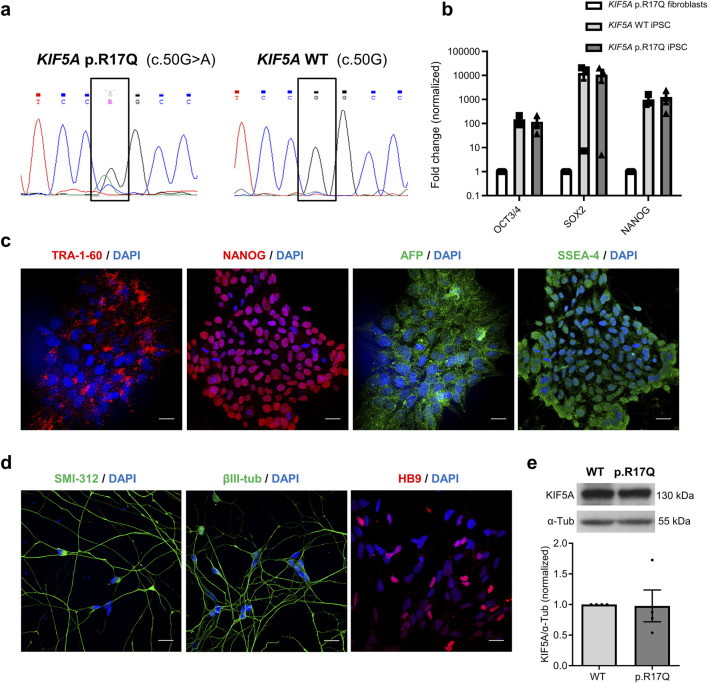
Characterization of the *KIF5A* mutant (p.R17Q) and isogenic wild-type (WT) iPSCs and iPSC-MNs. **(a)** Sanger sequencing electropherograms of the *KIF5A* p.R17Q and of the corrected WT iPSC lines. **(b)** Gene expression analysis of the stemness transcription factors *OCT3/4*, *SOX2*, and *NANOG* in the original SPG10 patient’s fibroblasts with the *KIF5A* p.R17Q variant and in *KIF5A* WT and p.R17Q iPSC lines by Q-PCR. Mean ± SEM; non-parametric Mann–Whitney U test; n = 3 different cell passages in culture. **(c)** Representative confocal images of *KIF5A* iPSCs stained for the stemness markers TRA1-60 and NANOG (red), AFP (green), and SSEA-4 (green). Cell nuclei were stained with DAPI (blue). Scale bar, 20 μm **(d)** Representative confocal images of the neuronal markers SMI-312 and βIII-tubulin (green) and of the motoneuronal marker HB9 (red) in differentiated *KIF5A* iPSC-MNs. Cell nuclei were stained with DAPI (blue). Scale bar, 10 μm. **(e)** Representative Western blot images of KIF5A protein in *KIF5A* WT and p.R17Q iPSC-MNs (above) and their densitometric analysis (below). Mean ± SEM; non-parametric Mann–Whitney U test, *p < 0.05; n = 4 independent iPSC-MN differentiations/line.

The novel N-terminal *KIF5A* VUS (p.R17Q), which we recently identified in a SPG10 pedigree and predicted to impair ATP/ADP binding in the motor domain, was corrected in the iPSC line we recently generated and characterized from one out of the three affected members (Santangelo et al., 2023). By CRISPR/Cas9 gene-editing, we obtained the isogenic *KIF5A* WT iPSC line, as confirmed by Sanger sequencing (Figure 1a).

Cytogenetic analysis by Q-banding showed a normal karyotype and no gross rearrangements in the original mutant *KIF5A* p.R17Q iPSCs, as already reported (Santangelo et al., 2023), and in its gene-edited derivative WT cell line (data not shown). Both the *KIF5A* iPSC lines tested positive for the stemness markers *OCT3/4*, *SOX2*, and *NANOG* by Q-PCR (Figure 1b), and TRA-1–60, NANOG, AFP (Alpha-Fetoprotein), and SSEA-4 by IF (Figure 1c) confirming their pluripotency features.

We differentiated the two *KIF5A* iPSC lines into iPSC-MNs following a 34 days protocol and confirmed they were positive for the neuronal cytoskeletal markers SMI-312 and βIII-tubulin and the motoneuronal marker HB9 (Figure 1d). The differentiation efficiency was evaluated by Q-PCR of the neuronal markers *TUBB3* (βIII-tubulin) and *MAP2*, as well as of the motoneuronal marker *ChAT*. Our results showed no differences in gene expression levels of both neuronal and motoneuronal markers between the mutant p.R17Q and the WT KIF5A iPSC-MNs in three differentiation replicates (Supplementary Figure S1). Analysis of KIF5A protein levels by Western blot (WB) revealed no difference between the mutant *KIF5A* p.R17Q and the isogenic WT iPSC-MNs (Figure 1e).

### Mutant KIF5A p.R17Q protein distribution and axonal swellings in iPSC-MNs

**FIGURE 2:**
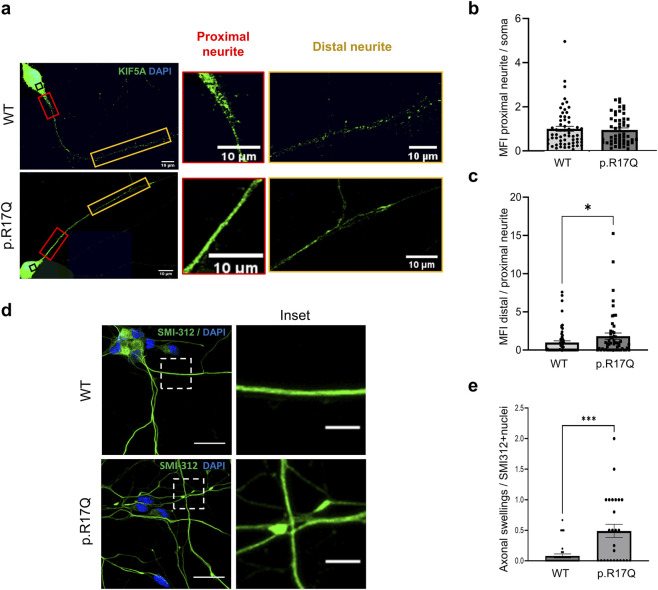
Analysis of KIF5A distribution and axonal swellings in *KIF5A* iPSC-MNs. **(a)** Staining of KIF5A protein (green) in *KIF5A* WT and mutant p.R17Q iPSC-MNs by IF. Cell nuclei were stained with DAPI (blue). Scale bar, 10 μm. KIF5A distribution was analyzed in three different neuronal areas: the soma cytoplasm (black rectangle), the neurite segments proximal to the soma (red rectangle), and the distal neurite segments (∼50 μm from the soma; yellow rectangle). **(b)** Ratio of KIF5A mean fluorescence intensity (MFI) in proximal neurite segments over the soma and **(c)** ratio of KIF5A MFI in distal neurite over proximal neurite segments. Data were normalized on the WT iPSC-MN line. Mean ± SEM; non-parametric Mann–Whitney U test, *p < 0.05; n = 3 independent iPSC-MNs differentiations/line; >30 cells/line were analyzed in the three replicates. **(d)** Representative confocal images of SMI-312 marker (green) in *KIF5A* WT and p.R17Q iPSC-MNs. Cell nuclei were stained with DAPI (blue). Full image (left), scale bar, 10 μm; inset magnification (right), scale bar, 5 μm. **(e)** Quantitative analysis of axonal swellings normalized over the number of nuclei of SMI-312-positive (+) iPSC-MNs. Mean ± SEM; non-parametric Mann–Whitney U test, **p < 0.01, *p < 0.05; n = 3 independent iPSC-MN differentiations/line; >25 cells/line were analyzed in the three replicates.

Since KIF5A is responsible for the axonal anterograde transport acting in dimers, we investigated whether the p.R17Q variant, while not affecting KIF5A protein levels, might influence kinesin motor activity and indirectly its distribution along neurites. For this purpose, we performed IF staining and measured the KIF5A protein mean fluorescence intensity (MFI) in three different motoneuronal cell regions: the soma cytoplasm, the neurite segments proximal to the soma, and the distal neurite segments (∼50 μm distance from the soma) (Figure 2a). To evaluate KIF5A protein redistribution from the cell soma into neurites, we first calculated the ratio between the KIF5A MFI in proximal neurite segments and in the soma. No statistically significant differences in KIF5A protein distribution in proximal neurites were observed between the two *KIF5A* iPSC-MN lines (Figure 2b).

To assess the ability of the mutant *KIF5A* protein to move and distribute from proximal to distal neurites, we determined the ratio between KIF5A MFI in neurite segments distal and proximal to the soma. We observed a significant increased ratio in the mutant *KIF5A* iPSC-MNs compared to the WT ones (Figure 2c), likely indicating an impaired transport and accumulation of the mutant protein in more distal neurites.

To further assess possible axonal transport defects, we analyzed the presence of axonal swellings by performing IF staining for SMI-312, an axonal marker of highly phosphorylated medium and heavy neurofilaments (Figure 2d). We observed a significant increase in the number of neurofilament-positive axonal swellings in the mutant *KIF5A* p.R17Q iPSC-MNs compared to the WT iPSC-MNs (Figure 2e). By a parallel labelling with Mitotracker Red CMXRos dye, we representatively showed that mitochondria were also trapped at axonal swellings in the *KIF5A* p.R17Q iPSC-MNs (Supplementary Figure S2).

### Analysis of mitochondria and lysosome transport in mutant *KIF5A* iPSC-MNs

**FIGURE 3:**
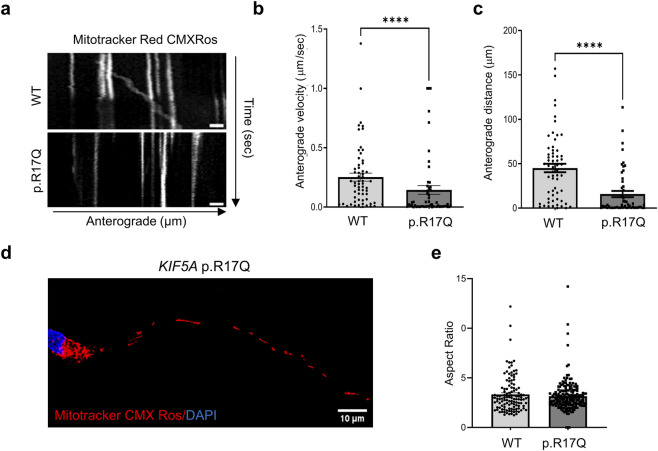
Mitochondrial transport in *KIF5A* iPSC-MNs. **(a)** Representative kymographs of Mitotracker Red CMXRos-labelled mitochondrial movement in *KIF5A* WT and p.R17Q iPSC-MNs. The y-axis shows the travel time along neurites (seconds), while the x-axis shows the distance traveled (μm). Scale bar, 10 μm. Quantitative analysis of **(b)** the mitochondria mean anterograde velocity (μm/sec) and **(c)** the distance (μm) traveled by motile mitochondria in *KIF5A* WT and p.R17Q iPSC-MNs. Mean ± SEM; non-parametric Mann–Whitney U test, ****p < 0.0001; n = 3 independent iPSC-MN differentiations/line, >60 mitochondria/cell line were analyzed in the three replicates. **(d)** Representative confocal images of mitochondria labeled with Mitotracker Red CMXRos in the *KIF5A* p.R17Q iPSC-MNs. Cell nuclei stained with DAPI (blue). Scale bar, 10 μm. **(e)** Quantification of the mitochondrial Aspect Ratio, calculated as mitochondria width/height in *KIF5A* WT and p.R17Q iPSC-MNs. Mean ± SEM; non-parametric Mann–Whitney U test; n = 3 independent iPSC-MN differentiations/line; >50 mitochondria/cell line were analyzed in the three replicates.

To examine the impact of the *KIF5A* p.R17Q VUS on mitochondrial transport, we performed a live cell time-lapse assay after mitochondria labelling with the Mitotracker dye. The representative kymographs for both the mutant and the WT *KIF5A* iPSC-MN lines are shown (Figure 3a). We analyzed the mean mitochondrial anterograde velocity and the distance traveled by motile mitochondria in selected neurites regions as detailed in Materials and Methods, focusing on anterogradely moving mitochondria. The results showed a significant decrease in both mean anterograde velocity (Figure 3b) and distance travelled by the mitochondria (Figure 3c) in *KIF5A* p.R17Q iPSC-MNs compared to the WT iPSC-MNs.

To investigate whether the observed changes in mitochondrial anterograde transport also affected mitochondrial morphology (Figure 3d), we calculated the Aspect Ratio (AR) parameter related to the mitochondria along the analyzed neuritic processes. AR did not significantly differ between the two *KIF5A* iPSC-MN lines (Figure 3e).

**FIGURE 4:**
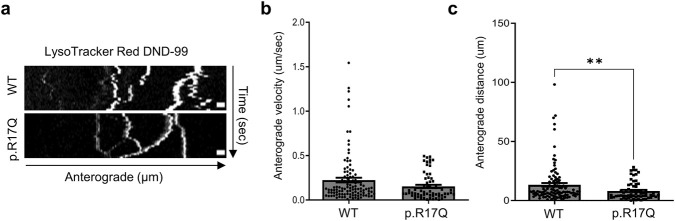
Lysosomal transport in *KIF5A* iPSC-MNs. **(a)** Representative kymographs of LysoTracker-labelled lysosomal movement in *KIF5A* WT and p.R17Q iPSC-MNs. The y-axis shows the travel time along neurites (seconds), while the x-axis the shows the distance traveled (μm). Scale bar, 10 μm. Quantitative analysis of the **(b)** lysosomal mean anterograde velocity (μm/sec) and **(c)** distance (μm) traveled by motile lysosomes in *KIF5A* WT and p.R17Q iPSC-MNs. Mean ± SEM; non-parametric Mann–Whitney U test, ****p < 0.0001, *p < 0.05; n = 3 independent iPSC-MN differentiations/line; >50 lysosomes/cell line were analyzed in the three replicates.

To evaluate the transport of other cargoes, such as lysosomes, *KIF5A* iPSC-MNs were incubated with the LysoTracker Red DND-99 dye. Lysosomal movement was assessed by time-lapse imaging and representative kymographs were generated for both the mutant and the WT *KIF5A* iPSC-MN lines (Figure 4a). We analyzed the mean anterograde velocity and the distance travelled by motile lysosomes in specific neurites regions, focusing on anterogradely moving lysosomes. Our results showed a trend towards a reduced velocity in mutant p.R17Q iPSC-MNs compared to the WT cells (Figure 4b). Consistent with the reduced velocity, the lysosome travel distance was significantly reduced in the mutant *KIF5A* p.R17Q iPSC-MNs compared to the WT cells (Figure 4c).

## Discussion

Mutations in *KIF5A* gene cause a range of neurodegenerative and neurodevelopmental disorders where the clinical phenotype is strongly associated with two distinct mutational hotspots. Mutations in *KIF5A* N-terminal motor and stalk domains are linked to SPG10 (Reid et al., 2002; Jennings et al., 2017) or CMT2 (Crimella et al., 2012), while variants in the cargo-binding domain in the tail region are linked to ALS (Brenner et al., 2018; Nicolas et al., 2018) and NEIMY (Duis et al., 2016). This spectrum of clinical presentations, along with their link with mutations in distinct KIF5A protein domains, suggest the presence of different underlying biological mechanisms driving this phenotypic variability which need to be further investigated.

In a recent mutational screening of a large cohort of Italian patients with different *KIF5A*-related disorders, we reported the novel p.R17Q VUS in a SPG10 pedigree segregating with disease in three family members. This variant is phylogenetically conserved and predicted to disrupt ATP/ADP binding in the kinesin motor domain (Cozzi et al., 2024), likely impairing axonal transport function. Building on this, we previously generated an iPSC line from one member of this *KIF5A*-mutated SPG10 pedigree (Santangelo et al., 2023). Being the p.R17Q a VUS, we aimed to functionally assess the pathogeneicity of this variant by studying its impact on KIF5A protein sub-cellular distribution and axonal transport in iPSC-derived motoneurons. We here therefore developed the corresponding isogenic WT iPSC line using the CRISPR/Cas9 system, allowing us to study the single p.R17Q variant in the same genetic background.

Our results show that the *KIF5A* p.R17Q VUS did not affect MN differentiation efficiency. However, given a certain variability observed among replicates, we can not completely assess if this result reflects the intrinsic variability of the small molecules-based differentiation protocol or a causal effect of the mutant KIF5A protein. Moreover, the KIF5A p.R17Q VUS did not determine a significant change in KIF5A protein levels in iPSC-MNs. This observation differs from previous findings we obtained in murine NSC-34 cells where KIF5A p.R17Q showed reduced levels, likely due to protein instability (Cozzi et al., 2024). This discrepancy may reflect differences in the two experimental cell models used with mutant *KIF5A* overexpression in the NSC-34 cell line and its physiological expression here in human iPSC-MNs.

When we examined the intracellular distribution of the mutant KIF5A motor protein, the use of iPSC-MNs enabled an accurate quantification of KIF5A levels, specifically along neurites. We found KIF5A p.R17Q localization was similar at short distance from the cell soma compared to the WT kinesin, while it was significantly increased in the mutant KIF5A p.R17Q iPSC-MNs at more long distance neurite regions. This observation might suggest motor defects favouring an abnormal accumulation of the mutant KIF5A protein along neurites.

To support this hypothesis, we preliminarily investigated the presence of axonal swellings, a hallmark of cytoskeletal defects and abnormal cargo accumulation, already observed in neurodegenerative diseases (Khalil et al., 2020). In a *Drosophila* model of SPG10, the mutant *KIF5A* p.N262S kinesin was reported to disrupt axonal transport and to induce axonal swellings containing multivesicular bodies, dark pre-lysosomal vacuoles, autophagosomes, and lysosomal organelles (Füger et al., 2012). Our results confirmed an increase of neurofilament-positive axonal swellings in the mutant *KIF5A* p.R17Q iPSC-MNs, which we showed contained also mitochondria. Moreover, both the genetic deletion of *KIF5A* (Uchida et al., 2009) and the ectopic expression of the SPG10-associated *KIF5A* p.N256S (Wang and Brown, 2010) were already shown to reduce the anterograde and retrograde transport of neurofilaments in mouse primary cortical neurons. The accumulation of neurofilaments and mitochondria observed in our experimental disease model suggests an impaired axonal transport, as KIF5A is known to regulate the trafficking of both these cargoes (Cozzi et al., 2025).

Consistent with a KIF5A-related impaired transport, we found that the mutant p.R17Q *KIF5A* also causes a significant reduction in both anterograde transport velocity and distance traveled by mitochondria in iPSC-MNs. Similarly, *KIF5A*-null NGN2-inducible iPSC-MNs exhibited reduced anterograde velocity and increased stalling of mitochondria (Guerra San Juan et al., 2025), further supporting the pivotal role of KIF5A in neurons. Additionally, our observation that the shape of transported mitochondria was unaffected by the expression of the mutant *KIF5A* p.R17Q is in line with previous studies performed on *Drosophila* larvae expressing the distinct, SPG10-linked, *KIF5A* p.N262S mutation (Füger et al., 2012).

A previous work investigating lysosomal transport showed that trimethyltin chloride-induced *KIF5A* downregulation disrupted lysosomes trafficking in mouse hippocampal neurons, while its overexpression rescued their transport and reduced neurotoxicity (Liu et al., 2021). Our results confirmed reduced anterograde lysosomal velocity and distance traveled by lysosomes along neurites in the mutant *KIF5A* p.R17Q iPSC-MNs, indicating impaired transport of different organelles as a consequence of mutant KIF5A dysfunction.

Indeed, we showed that the p.R17Q VUS, mapping in the ATP-binding pocket and predicted to affect kinesin motor activity, results in slower movement and shorter transport distances of both mitochondria and lysosomes cargoes, thereby functionally proving the pathogenic nature of this variant. The formation of dysfunctional KIF5 dimers, either with one mutant or two mutant KIF5A subunits, which likely stall on microtubules and create local traffic jams and swelling, is able to impair proper cargo transport by a likely dominant-negative effect. On the contrary, the ALS-associated frameshifts mutations in the C-terminal tail act with a gain-of-function mechanism by causing the disruption of the autoinhibitory IAK motif, which determines hyperactivation and dysfunction of KIF5A, leading to an increased mitochondrial transport (Baron et al., 2022).

In conclusion, in this study we provide functional evidence that the novel p.R17Q VUS in *KIF5A,* by impairing axon health and cargo transport, should be considered pathogenic, thereby extending the spectrum of *KIF5A* mutations causing SPG-10. Moreover, we support that patient-derived and gene-edited iPSC-MNs are suitable *in vitro* tools for VUS validation and for a better understanding of the pathomechanisms underlying KIF5A-related clinical heterogeneity.

## Data Availability

The datasets presented in this study can be found in the online repository Zenodo. The accession link can be found below: https://doi.org/10.5281/zenodo.16086281.

## Glossary

aCSF: artificial cerebrospinal fluid
AFP: Alpha-Fetoprotein
ALS: amyotrophic lateral sclerosis
AR: Aspect Ratio
BSA: Bovine Serum Albumin
CMT2: Charcot-Marie-Tooth Type 2
DAPI: 4′,6-diamidino-2-phenylindole
EBs: embryoid bodies
HDR: Homology Directed Repair
IAK: isoleucine-alanine-lysine
IF: immunofluorescence
iPSC-MNs: iPSC-motoneurons
iPSCs: induced-pluripotent stem cells
KHC: kinesin heavy chain
MFI: Mean fluorescence intensity
MOPS: 3-N-morpholino propanesulfonic acid
NEIMY: neonatal intractable myoclonus
OMIM: Online Mendelian Inheritance in Man
PBS: phosphate buffered saline
Q-PCR: Quantitative PCR
RH: Relative Humidity
RNP: ribonucleoprotein
ROI: region of interest
RT: room temperature
SEM: standard error mean
sgRNA: single-guide RNA
SPG10: hereditary spastic paraplegia 10
ssODN: single-stranded-oligodeoxynucleotide
TBST: Tris-Buffered Saline with Tween-20
VUS: variant of unknown significance
WB: western blot
