# Supplementary material for: Functional validation of the novel KIF5A p.R17Q VUS reveals defective axonal transport in iPSC-motoneurons from a SPG10 patient
Source: Front Genet. 2026 Feb 27;17:1774170. doi: 10.3389/fgene.2026.1774170 (PMC12981723; doi:10.3389/fgene.2026.1774170)
Supplement: Supplementary file 1 [file DataSheet1.pdf]

Supplementary materials

Table S1: List of primary and secondary antibodies used for immunofluorescence (IF) and Western blot (WB) assays

**Primary Antibody**	**Company and Catalog #**	**Use**	**Dilution**
TRA-1-60	Invitrogen (14-8863-80)	IF	1:125
NANOG	Cell Signaling (3580)	IF	1:500
AFP (Alpha-Fetoprotein)	Abcam (ab108337)	IF	1:250
SSEA-4	Invitrogen (14-8843-80)	IF	1:100
HB9	Invitrogen (PA5-23407)	IF	1:200
Beta III-Tubulin	Abcam (ab52623)	IF	1:500
SMI-312	Covance (SIG-32248)	IF	1:5000
KIF5A	Abclonal (A3303)	IF	1:100
KIF5A	Abclonal (A3303)	WB	1:500
Tubulin	Sigma-Aldrich (T6199)	WB	1:1000
**Secondary Antibody**			
Alexa Fluor 488 anti-mouse	Invitrogen (A11034)	IF	1:500
Alexa Fluor 555 anti-rabbit	Thermo Fisher Scientific (A21430)	IF	1:500
Anti-mouse IgG- HRP	Sigma-Aldrich (A4416)	WB	1:20000
Anti-rabbit IgG- HRP	Cell Signaling (7074S)	WB	1:2000

Table S2: Off-target prediction by IDT

#MM, mismatch number

Sequence	PAM	Score	#MM	Gene	Locus
TCAAGGTGCTCTGCCGATTC	CGG	N/A		KIF5A	chr12:+57550299
TCAAGGC-CTCTGCCAATTC	CAG	32	3		chr8:-108508479
TCAATGTGCTCTGCC-ATTC	AGG	42	2		chr5:+139308664
TCTGGGAGCTCTGCAGATTC	AGG	51	4		chr12:-131274097
CCCAGGTCCTCTGCCCATTC	GGG	53	4		chr3:+127194880
TCAGTGTGCTGTGCCAATTC	CAG	59	4		chr13:-44282757
TCAAGGA-CTCTGCCCATTC	TAG	60	3		chr8:-63060
TCAAGGA-CTCTGCCCATTC	TAG	60	3		chr19:-259211
TCAAGGA-CTCTGCCCATTC	TAG	60	3		chr18:-101245
TCAAGGA-CTCTGCCCATTC	TAG	60	3		chr6:-151634
TCAAGGA-CTCTGCCCATTC	TAG	60	3		chr2:-113620903

Supplementary Figures

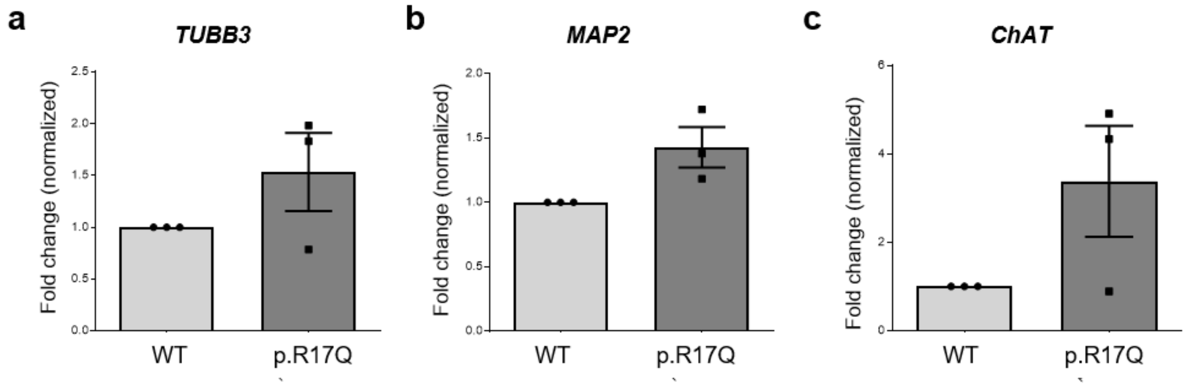

Figure S1: Gene expression analysis of neuronal and motoneuronal markers in *KIF5A* iPSC-MN lines.

Gene expression analysis of the neuronal markers **a)** βIII-tubulin (*TUBB3*) and **b)** *MAP2* and **c)** of the motoneuronal marker *ChAT* in *KIF5A* WT and p.R17Q iPSC-MNs by Q-PCR, expressed as fold change values. Mean±SEM; non-parametric Mann–Whitney U test; n=3 independent iPSC-MNs differentiation/line.

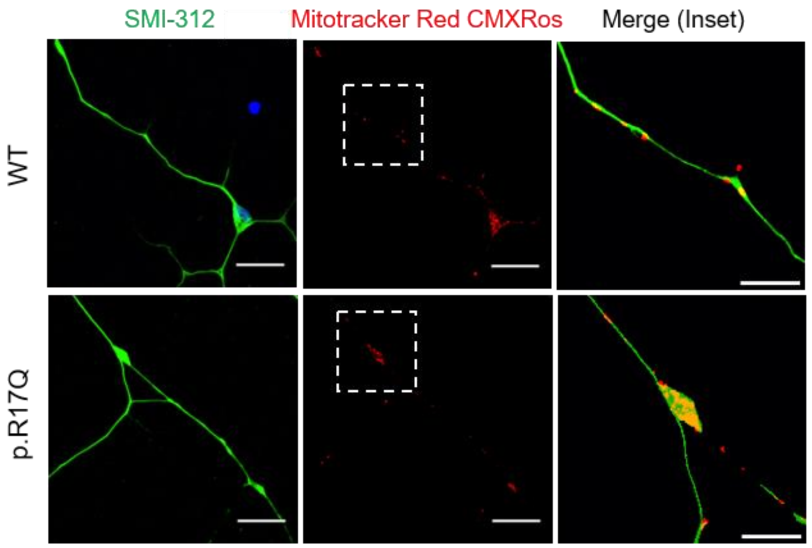

Figure S2: Representative confocal images of SMI-312 (green) and Mitotracker Red CMXRos dye (red) (scale bar, 20 μm) in the two KIF5A iPSC-MNs and merge images of the magnification inset (scale bar, 10 μm).

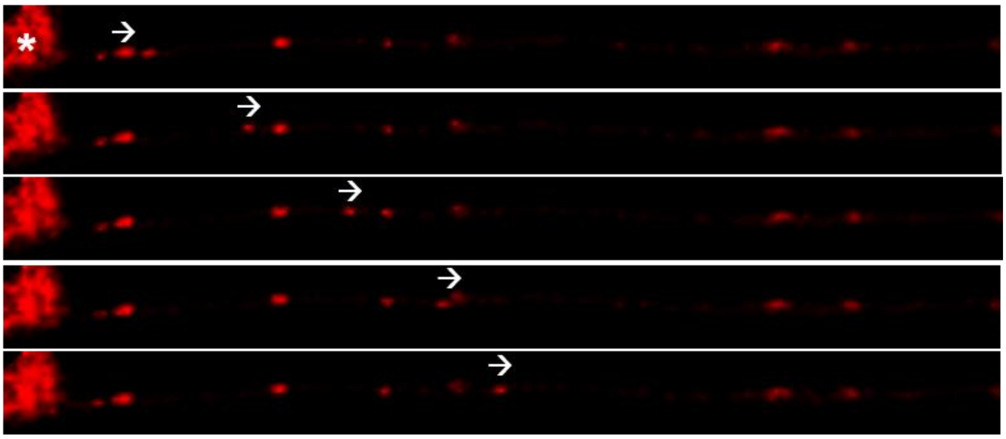

Figure S3: Representative series of confocal images of the movement trajectory of a Mitotracker Red CMXRos-labelled mitochondrion in *KIF5A* WT iPSC-MNs using the ImageJ MTrackJ plugin, tracking with an asterisk its movement over time (1 frame every two seconds for 3 minutes).
